# Comparison of COVID-19 Pandemic Waves in 10 Countries in Southern Africa, 2020–2021

**DOI:** 10.3201/eid2813.220228

**Published:** 2022-12

**Authors:** Joshua Smith-Sreen, Bridget Miller, Alinune N. Kabaghe, Evelyn Kim, Nellie Wadonda-Kabondo, Alean Frawley, Sarah Labuda, Eusébio Manuel, Helga Frietas, Anne C. Mwale, Tebogo Segolodi, Pauline Harvey, Onalenna Seitio-Kgokgwe, Alfredo E. Vergara, Eduardo S. Gudo, Eric J. Dziuban, Naemi Shoopala, Jonas Z. Hines, Simon Agolory, Muzala Kapina, Nyambe Sinyange, Michael Melchior, Kelsey Mirkovic, Agnes Mahomva, Surbhi Modhi, Stephanie Salyer, Andrew S. Azman, Catherine McLean, Lul P. Riek, Fred Asiimwe, Michelle Adler, Sikhatele Mazibuko, Velephi Okello, Andrew F. Auld

**Affiliations:** Public Health Institute/US Centers for Disease Control and Prevention Global Health Fellowship Program, Lilongwe, Malawi (J. Smith-Sreen, B. Miller);; Malawi Centers for Disease Control and Prevention, Lilongwe (A.N. Kabaghe, E. Kim, N. Wadonda-Kabondo, A.F. Auld);; Angola Centers for Disease Control and Prevention, Luanda, Angola (A. Frawley, S. Labuda);; Angola Ministry of Health, Luanda (E. Manuel, H. Frietas);; Public Health Institute of Malawi (PHIM), Lilongwe (A.C. Mwale);; Botswana Centers for Disease Control and Prevention, Gaborone, Botswana (T. Segolodi, P. Harvey);; Botswana Ministry of Health and Wellness, Gaborone (O. Seitio-Kgokgwe);; Mozambique Centers for Disease Control and Prevention, Maputo, Mozambique (A.E. Vergara);; Mozambique National Institute for Health, Maputo (A.E. Vergara, E.J. Gudo);; Namibia Centers for Disease Control and Prevention, Windhoek, Namibia (E.J. Dziuban);; Namibia Ministry of Health and Social Services, Windhoek (N. Shoopala); Zambia; Centers for Disease Control and Prevention, Lusaka, Zambia (J.Z. Hines, S. Agolory);; Zambia National Public Health Institute (ZNPHI), Lusaka (M. Kapina, N. Sinyange);; Zimbabwe Centers for Disease Control and Prevention, Harare, Zimbabwe (M. Melchior, K. Mirkovic);; Zimbabwe Office of the President and Cabinet, Harare (A. Mahomva);; US Centers for Disease Control and Prevention, Atlanta, Georgia, USA (S. Modi, S.J. Salyer, C. McLean);; Africa Centres for Disease Control and Prevention, Addis Ababa, Ethiopia (S.J. Salyer, L.P. Riek);; Johns Hopkins Bloomberg School of Public Health, Baltimore, Maryland, USA (A.S. Azman);; Lesotho Centers for Disease Control and Prevention, Maseru, Lesotho (F. Asiimwe);; Eswatini Centers for Disease Control and Prevention, Lobamba, Swaziland (M. Adler, S. Mazibuko);; Eswatini Ministry of Health, Lobamba (V. Okello); The Global Fund, Geneva, Switzerland (A.F. Auld)

**Keywords:** COVID-19, SARS-CoV-2, coronavirus disease, severe acute respiratory disease coronavirus 2, viruses, respiratory infections, COVID-19 testing, comparative studies, Africa, southern Africa

## Abstract

We used publicly available data to describe epidemiology, genomic surveillance, and public health and social measures from the first 3 COVID-19 pandemic waves in southern Africa during April 6, 2020–September 19, 2021. South Africa detected regional waves on average 7.2 weeks before other countries. Average testing volume 244 tests/million/day) increased across waves and was highest in upper-middle-income countries. Across the 3 waves, average reported regional incidence increased (17.4, 51.9, 123.3 cases/1 million population/day), as did positivity of diagnostic tests (8.8%, 12.2%, 14.5%); mortality (0.3, 1.5, 2.7 deaths/1 million populaiton/day); and case-fatality ratios (1.9%, 2.1%, 2.5%). Beta variant (B.1.351) drove the second wave and Delta (B.1.617.2) the third. Stringent implementation of safety measures declined across waves. As of September 19, 2021, completed vaccination coverage remained low (8.1% of total population). Our findings highlight opportunities for strengthening surveillance, health systems, and access to realistically available therapeutics, and scaling up risk-based vaccination.

As of September 2021, in Africa, 5,650,962 SARS-CoV-2 infections (2.6% of global total) and 135,568 related deaths (3.0% of global total), had been reported (*1*). However, this number was likely a substantial underestimate of the true number of SARS-CoV-2 infections, given limited surveillance capacity and relatively higher positivity reported in seroprevalence studies (*2*–*4*). The first case in southern Africa, home to ≈14% of the population of Africa (*5*), was reported on March 5, 2020 in South Africa (*6*). By September 2021, all countries in southern Africa were experiencing their third COVID-19 pandemic waves.

Although quantitative comparisons of COVID-19 waves have been published, few have compared waves in southern Africa (*7*–*9*), despite the region experiencing substantial illness and death across waves (*10*). Furthermore, there has been limited systematic reporting and analysis of public health and social measures (PHSMs) enacted during outbreaks across countries in the region. A comparison of characteristics across waves provides unique insights into reported incidence, mortality, and distribution of variants of concern (VOCs) across geography and time. Population movements between countries in southern Africa, a highly interconnected region, have historically been drivers of HIV and tuberculosis epidemics (*11*) and could influence COVID-19 wave propagation. To inform public health actions to prevent, detect, and reduce the effects of future COVID-19 pandemic waves across the region, we compared trends in reported testing volume, incidence, mortality, genomic surveillance results, PHSMs, and vaccination coverage across pandemic waves in southern Africa during April 2020–September 2021.

## Methods

### Data Sources and Data Collection

According to the African Union (https://au.int), southern Africa consists of Angola, Botswana, Eswatini, Lesotho, Malawi, Mozambique, Namibia, South Africa, Zambia, and Zimbabwe. We obtained data on testing, incidence, mortality, and vaccination collected during February 7, 2020–September 19, 2021 (final day of data extraction) from the Our World in Data (OWID; https://ourworldindata.org) dataset, compiled by Johns Hopkins University (*1*). We supplemented missing data or errors with data from in-country US Centers for Disease Control and Prevention (CDC) offices, the World Health Organization (WHO), or daily reports from Africa Centers for Disease Control and Prevention (Africa CDC) (*12*). We excluded still-missing data from indicator computations and computed weekly averages for each indicator to reduce potential bias introduced by missed reports. We based the effective reproduction number on estimates published elsewhere (*13*). We obtained publicly available SARS-CoV-2 genomic sequencing results from GISAID (https://www.gisaid.org) (*14*); those data were exported on September 19, 2021, and included specimens collected during March 1, 2020–September 6, 2021.

We extracted publicly available PHSM data from the Oxford COVID-19 Government Response Tracker (OxCGRT; https://covidtracker.bsg.ox.ac.uk), available during January 1, 2020–September 19, 2021 (*15*). OxCGRT contains 23 indicators aggregated into a set of common indices, rated 1–100 to quantify the level of government intervention. All indices, defined on the OxCGRT website, were based on averages of component indicators to provide a measure of how many indicators a government has acted upon and to what degree. We compared the original PHSM stringency, overall government response, containment health, and economic support indices across waves. This activity was reviewed by CDC and conducted consistent with applicable federal laws and CDC policy.

### Statistical Analysis

To align with existing analysis of pandemic waves in Africa, we adapted wave definitions published elsewhere (*6*) (Appendix). Different authors independently applied these definitions to determine the wave start, peak, and end weeks (Appendix Table 1); we resolved discrepancies by consensus. We analyzed data in R version 4.01 (The R Foundation for Statistical Computing, https://www.r-project.org). We computed averages and maximums across wave periods and countries for reported COVID-19 incidence (7-day average daily cases and peak cases/1 million persons); mortality (7-day average daily deaths and peak deaths/1 million persons and case-fatality ratio [CFR]); testing (7-day average daily tests/1 million persons, 7-day average test positivity, peak 7-day average test positivity, and 7-day average tests per case); and vaccination (total number of persons vaccinated/100 population, total number persons fully vaccinated/100 persons [defined by OWID as total number of persons who received all doses prescribed by the vaccination protocol/100 persons in the total population], and average weekly vaccinations/1 million persons). We computed peak averages as the maximum 7-day average in a period; OWID defines peak 7-day average test positivity as tests conducted per new confirmed case. We computed regional averages for southern Africa by averaging all available country-specific values for each indicator within the wave period. For example, for each 7-day average indicator, we averaged all available country-level 7-day averages to determine overall regional averages, and all available 7-day averages within country-specific wave periods were averaged for regional averages by wave. We conducted 1-way analysis of variance tests to calculate differences in 7-day average cases, deaths, and tests per 1 million persons across waves. We computed genomic surveillance coverage as the total number of sequences submitted to GISAID during that period divided by the number of cases per 1 million. However, for ease of interpretation, genomic surveillance coverage was reported as its inverse (number of cases/1 million/sequence submitted). Therefore, a country with a higher number of reported cases per 1 million per sample sequenced has lower genomic surveillance coverage than a country with a lower number. We computed medians and interquartile ranges (IQRs) across wave periods for continuous genomic variables and frequencies for categorical genomic variables. We reported genomic sequences using WHO genome labels (*16*) (Appendix Table 2) and categorized sequences without a WHO label as other lineages (Appendix Table 3). For PHSM data, we computed averages across waves for each index and frequencies for the number of measures mandated at the beginning, peak, end, and throughout the duration of waves.

## Results

### Burden of COVID-19 in Southern Africa

By September 19, 2021, southern Africa had 3,841,563 SARS-CoV-2 cases, 65.0% of Africa and 1.7% of global totals, and 107,347 COVID-19 deaths, 75.4% of Africa and 2.3% of global totals. South Africa had the highest numbers of cases (75.0%) and deaths (80.3%) among countries in the region. The countries with highest incidence and mortality over the period were Botswana, Namibia, Eswatini, and South Africa (Appendix Figure 1).

### Regional Pandemic Wave Propagation Patterns

**Figure 1:**
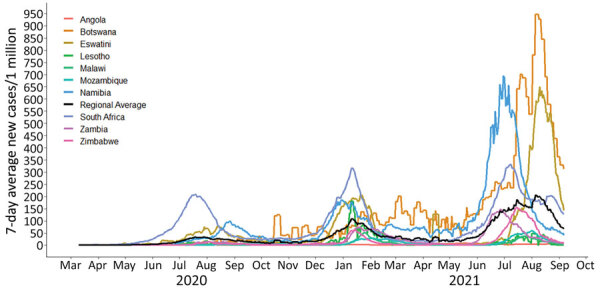
Reported 7-day average of new COVID-19 cases per 1 million population across 10 countries in southern Africa, March 5, 2020–September 17, 2021. Source: Our World in Data (https://www.ourworldindataorg), accessed 2021 Sep 20.

The earliest start date for the first wave within any country was April 6, 2020 (South Africa); by July 5, 2021, all countries in the region were experiencing a third wave (Figure 1). On average, pandemic waves in the region lasted 16.5 weeks; the first wave, at 19.5 weeks, was the longest, followed by the second, 15.1 weeks, and third, 14.9 weeks (Table 1). Wave durations varied by wave and across countries; the first wave in Angola lasted 30 weeks but the second wave in Zimbabwe lasted 9 weeks. Waves in almost all other countries started an average of 7.2 weeks later than in South Africa, but with some variation: Namibia at 4.0 weeks and Angola at 14.0 weeks later (Table 1).

**Table 1:** Total duration of 3 COVID-19 pandemic waves in 10 countries in southern Africa and time since start of wave in South Africa, April 6, 2020–September 19, 2021*

Country	Wave 1			Wave 2			Wave 3			Country average	
	Total duration, wk	Time from start of SA wave, wk		Total duration, wk	Time from start of SA wave, wk		Total duration, wk	Time from start of SA wave, wk		Total duration, wk	Time from start of SA wave, wk
Angola†	30	13		13	20		10	9		17.7	14.0
Botswana	31	5		16	8		17	2		21.3	5.0
Eswatini	15	11		14	4		10	9		13.0	8.0
Lesotho	16	11		12	3		14	5		14.0	6.3
Malawi	15	10		18	5		15	4		16.0	6.3
Mozambique	20	12		19	6		16	3		18.3	7.0
Namibia	20	10		13	0		17	2		16.7	4.0
SA	22	Referent		16	Referent		19	Referent		19.0	Referent
Zambia	15	13		21	3		17	2		17.7	6.0
Zimbabwe	11	12		9	7		14	5		11.3	8.0
Overall average	19.5	10.8		15.1	6.2		14.9	4.6		16.5	7.2

*Appendix* Table 1* shows dates of starts, peaks, ends, and period definitions of pandemic waves. SA, South Africa. *†Third wave in Angola had not yet reached its peak as of September 19, 2021.*

### Regional and Temporal Variations in Testing

The number of 7-day average daily tests per 1 million persons was higher in the 2 upper-middle-income countries, Namibia (549.0) and South Africa (519.3), where testing data were more available, but lower in low-income countries Malawi (37.9) and Mozambique (51.6) (Table 2). Testing increased in all 10 countries across successive pandemic waves; the third wave had nearly 3 times (388.0 vs. 146.8) the 7-day average daily tests per million persons than did the first wave. There was a statistically significant (p<0.05) mean difference across waves in tests within each country and across all countries. However, 7-day average test/case ratio was highest in the first wave (24.8), followed by the second (17.0) and third (13.5) (Table 2).

**Table 2:** Testing, illness, death, and vaccination comparison across 3 COVID-19 pandemic waves for 10 countries in southern Africa, March 5, 2020–September 19, 2021*

Country and wave	7-day averages and peaks								Peak Rt	Average CFR, %	Total vaccinated/100 pop	Total fully vaccinated/100 pop	7-d average no. vaccinations/1 million pop
	Average no. tests/1 million pop†	Average test positivity, %	Peak test positivity, %	Average no. tests/case	Average no. cases/1 million pop†	Peak no. cases/1 million pop	Average no. deaths/1 million pop†	Peak no. deaths/1 million pop					
Angola‡													
1	56.6	8.0	28.8	23.6	2.3	7.7	0.05	0.1	1.3	3.5	NA	NA	NA
2	70.4	8.5	34.0	16.8	5.1	8.6	0.11	0.3	1.2	2.3	2.8	1.6	421.6
3	85.9	6.5	17.2	20.8	4.9	9.5	0.18	0.3	1.2	2.5	5.4	2.9	455.3
Total	70.3	7.5	34.0	20.6	2.8	9.5	0.07	0.3	1.3	3.7	5.4	2.9	392.6
Botswana§													
1	NA	NA	NA	NA	27.5	124.8	0.08	0.3	1.2	0.5	NA	NA	NA
2	NA	NA	NA	NA	112.6	201.1	2.33	5.1	1.3	1.0	2.1	NA	597.5
3	NA	NA	NA	NA	397.7	946.6	5.25	16.0	1.4	1.5	15.3	9.3	1,751.2
Total	NA	NA	NA	NA	132.3	946.6	1.83	16.0	1.4	1.6	15.3	9.3	1,403.7
Eswatini§													
1	NA	NA	NA	NA	24.7	77.3	0.49	2.0	2.0	1.6	NA	NA	NA
2	NA	NA	NA	NA	87.7	205.9	4.40	14.0	1.8	3.1	NA	NA	NA
3	NA	NA	NA	NA	289.6	649.6	5.67	10.2	1.7	2.9	17.1	16.5	1,964.3
Total	NA	NA	NA	NA	70.1	649.6	1.85	14.0	2.0	2.5	17.1	16.5	1161.2
Lesotho§													
1	NA	NA	NA	NA	5.9	18.7	0.13	0.7	1.3	2.4	NA	NA	NA
2	NA	NA	NA	NA	46.0	180.7	1.36	4.8	1.5	2.0	NA	NA	NA
3	NA	NA	NA	NA	16.7	49.8	0.36	1.6	1.2	2.8	3.3	1.5	1,250.9
Total	NA	NA	NA	NA	13.6	180.7	0.38	4.8	1.5	2.5	3.3	1.5	743.3
Malawi¶													
1	17.6	8.9	24.8	22.3	1.9	5.6	0.06	0.3	1.8	2.7	NA	NA	NA
2	58.0	13.1	37.8	14.7	10.7	50.6	0.37	1.7	2.1	3.1	1.4	NA#	358.5
3	70.8	13.3	25.8	14.2	12.2	37.8	0.50	1.3	1.8	3.4	3.8	2.4	280.5
Total	37.9	9.2	37.8	31.8	5.9	50.6	0.22	1.7	2.1	3.5	3.8	2.4	266.9
Mozambique													
1	31.7	5.6	15.5	30.8	2.1	6.7	0.02	0.1	1.5	0.7	NA	NA	NA
2	60.4	16.3	33.6	9.8	11.7	29.7	0.15	0.5	1.7	1.1	0.2	NA#	169.7
3	97.0	18.6	35.9	11.0	20.7	58.9	0.28	0.8	1.8	1.2	5.8	4.9	702.2
Total	51.6	11.2	35.9	20.6	8.6	58.9	0.11	0.8	1.8	0.9	5.8	4.9	488.0
Namibia													
1	368.7	10.0	19.4	12.4	36.1	99.4	0.37	1.4	1.6	0.8	NA	NA	NA
2	590.1	13.6	25.7	10.3	88.2	185.6	0.98	2.4	1.5	1.0	NA	NA	NA
3	871.3	22.9	50.9	6.3	232.8	694.8	8.43	30.7	1.4	2.1	9.0	5.5	998.8
Total	549.0	12.7	50.9	23.9	88.9	694.8	2.43	30.7	1.6	1.3	9.0	5.5	776.5
South Africa													
1	392.8	12.5	27.8	14.9	63.3	209.6	1.55	5.0	1.5	1.9	NA	NA	NA
2	618.3	16.1	32.6	8.6	109.9	317.1	4.31	9.6	1.5	2.9	0.2	0.2	83.5
3	792.5	18.1	29.4	7.0	153.8	332.4	3.73	7.0	1.4	3.1	19.3	13.2	1,814.2
Total	519.3	13.1	32.6	13.2	85.6	332.4	2.55	9.6	2.6	2.6	19.3	13.2	1,205.7
Zambia													
1	107.5	9.9	30.1	32.5	6.1	15.9	0.13	0.7	1.1	2.4	NA	NA	NA
2	348.8	6.6	16.9	40.3	25.4	74.6	0.31	0.9	1.7	1.5	0.2	NA	85.1
3	387.3	11.0	26.0	21.3	48.6	147.5	1.00	3.2	1.8	1.6	1.6	1.5	227.2
Total	242.3	7.5	30.1	44.0	20.2	147.5	0.35	3.2	1.8	1.9	1.6	1.5	213.6
Zimbabwe													
1	52.7	6.5	21.3	37.3	3.7	13.3	0.11	0.4	1.8	2.4	NA	NA	NA
2	174.4	11.0	28.7	18.6	22.2	63.4	1.08	2.9	1.7	3.4	0.2	NA	118.8
3	411.1	11.2	22.8	14.2	55.7	156.1	1.86	5.2	1.9	3.5	19.5	13.7	2,437.0
Total	161.3	6.9	28.7	40.5	15.5	156.1	0.56	5.2	1.9	4.4	19.5	13.7	1,527.4
All#													
1	146.8	8.8	24.0	24.8	17.4	209.6	0.30	5.0	1.5	1.9	NA	NA	NA
2	274.3	12.2	29.9	17.0	51.9	317.1	1.54	14.0	1.6	2.1	NA	0.9	262.1
3	388.0	14.5	29.7	13.5	123.3	946.6	2.73	30.7	1.6	2.5	10.0	7.1	1,087.9
Total	243.9	10.0	35.7	28.3	44.8	322.7	1.05	8.7	1.8	2.5	10.8	8.1	832.3

*Source (*5*); accessed 2021 Sep 20. CFR, case-fatality ratio; NA, not available (either in the original dataset nor based on supplemental information) for this indicator in the given period; pop, population; Rt, effective reproduction number. †Variables on which a 1-way analysis of variance test was performed, and a significant difference in means was found between waves at α = 0.05 at a country level and across all countries. ‡Third wave was ongoing in Angola at the time of data extraction during epidemiologic week 90 (ending September 19, 2021). §These countries had no testing data in OWID. ¶These countries had no testing data in OWID, but testing data was provided by US Centers for Disease Control and Prevention collaborators, on the basis of nationally reported, publicly available aggregate data. #Regional averages for testing indicators were computed based on data from only 7 countries, excluding those for which testing data was not available (Botswana, Lesotho, Eswatini).

### Temporal Changes in COVID-19 Wave Severity

Average incidence (cases/1 million persons/day) increased across waves, from 17.4 in the first to 5.19 in the second 51.9 and 123.3 in the third. Percentage test positivity increased from 8.8% in the first wave to 12.2% in the second and 14.5% in the third. Mortality (deaths/1 million persons/day) increased from 0.3 in the first wave to 1.5 in the second and 2.7 in the third. CFR increased from 1.9% in the first wave to 2.1% in the second and 2.5% in the third (Table 2). 

**Figure 2:**
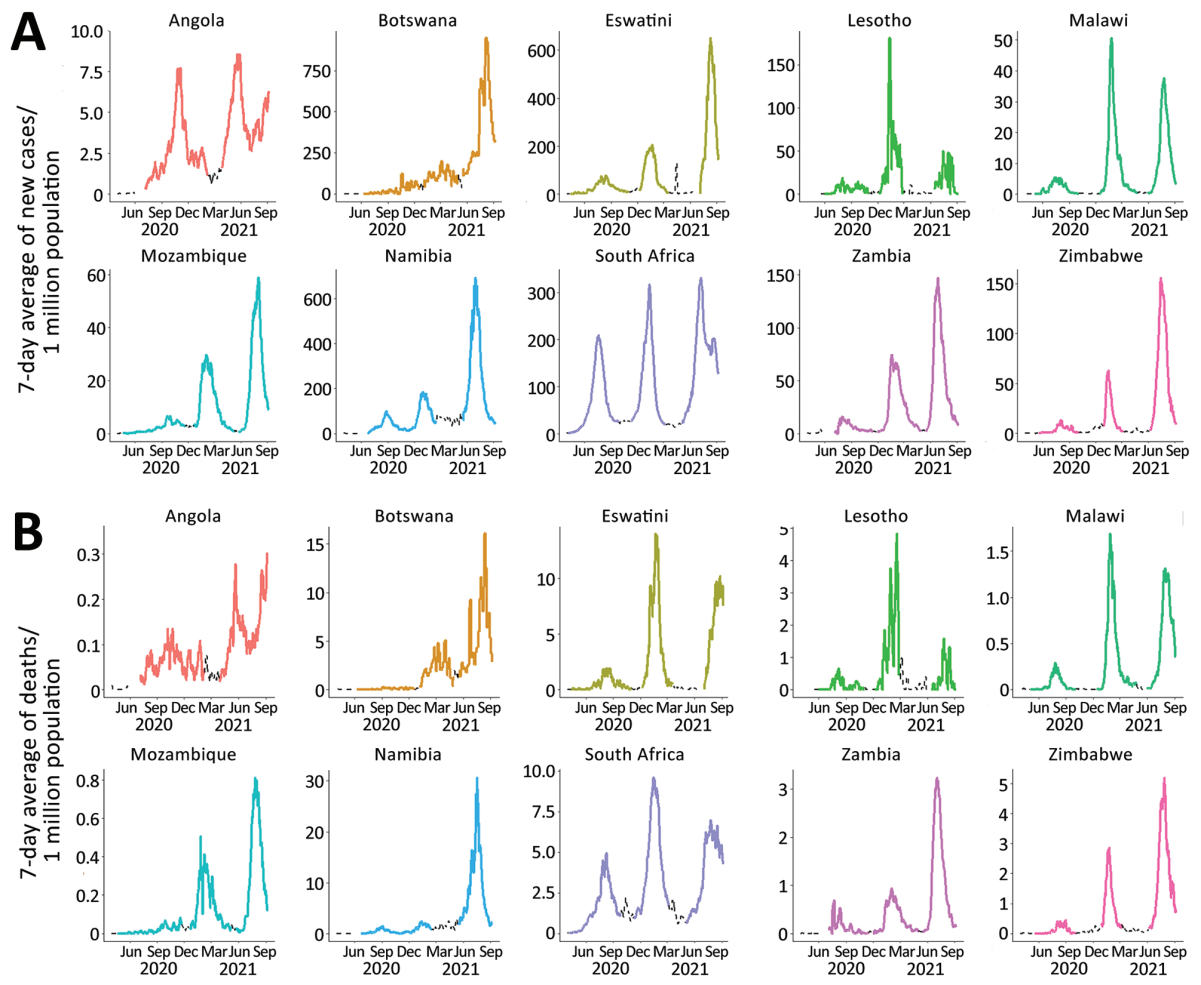
Reported 7-day average new COVID-19 cases (A) and deaths (B) per 1 million persons across pandemic waves in 10 countries in southern Africa, March 5, 2020–September 19, 2021. Colored lines indicate designated wave periods, dashed lines indicate periods between waves. We used differing y-axis scales in this figure to better visualize the wave patterns in each individual country. See Appendix Figure 2 for the same figure placed on corresponding y-axis scales to compare wave magnitudes across countries. Source: Our World in Data (https://www.ourworldindataorg), accessed 2021 Sep 20.

In an unadjusted analysis that did not control for changes in testing capacity over time, we also found a statistically significant (p<0.05) mean difference across waves in 7-day average daily cases and deaths per 1 million population within each country and the region. However, for some countries the second wave had the highest reported incidence of cases and deaths (Table 2; Figure 2). The second wave in Lesotho had the highest peak 7-day average number of new cases per 1 million persons and the highest peak in deaths per 1 million persons per day in Lesotho, South Africa, and Eswatini (Table 2). Upper middle-income countries South Africa, Namibia, and Botswana had relatively high overall 7-day average numbers of new deaths per 1 million persons compared with low-income countries.

### Genomic Surveillance

**Figure 4:**
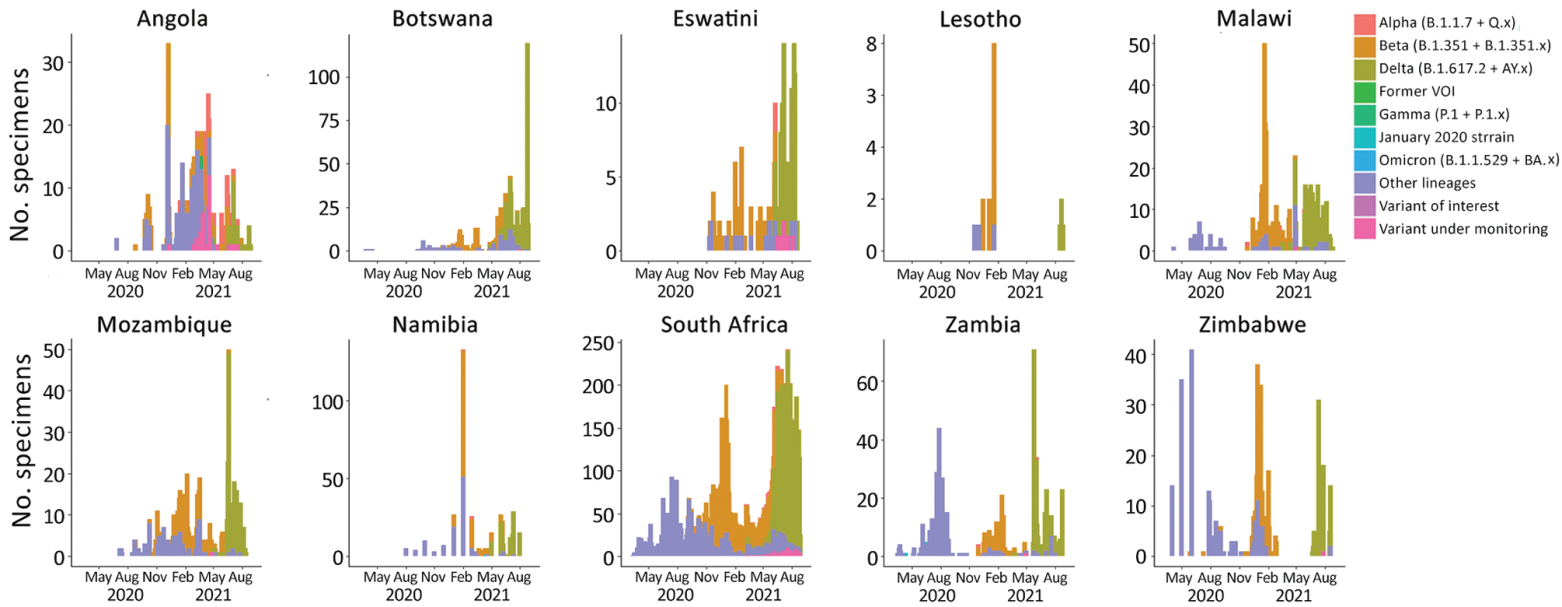
Counts of SARS-CoV-2 variants (World Health Organization classifications) in 10 countries in southern Africa, March 1, 2020–September 6, 2021. Definitions of variants are in Appendix Table. We used differing y-axis scales used in this figure to better visualize genomic sampling patterns in each individual country. See Appendix Figure 3 for the same figure placed on corresponding y-axis scales to compare wave magnitudes across countries. Source: GISAID (https://www.gisaid.org), accessed 2021 Sep 20.

During the study period, a collective 23,306 SARS-CoV-2 specimen sequences were submitted to GISAID from all 10 countries in southern Africa, most (89.4%) from laboratories in South Africa (Table 3; Appendix Figure 2). Most (18,464, 79.2%) specimens were collected in South Africa, the fewest (18, 0.1%) in Lesotho (Appendix Figure 3). The largest proportion of specimens (43.3%) were collected during the third wave; the number of sequences submitted increased between the first and second waves in 8/10 countries (Table 4; Figure 4).

**Table 3:** Overall genomic surveillance comparison across 3 COVID-19 pandemic waves for 10 countries in southern Africa, March 1, 2020–September 6, 2021*

Measure	Before wave 2 start	After wave 2 start, before wave 3 start	After wave 3 start	Overall
Total no. (%) specimens,†	5,543 (23.8)	7,660 (32.9)	10,103 (43.3)	23,306 (100)
Originating country, no. (%)				
Angola	615 (11.1)	264 (3.4)	20 (0.2)	899 (3.9)
Botswana	83 (1.5)	216 (2.8)	799 (7.9)	1098 (4.7)
Eswatini	11 (0.2)	77 (1.0)	34 (0.3)	122 (0.5)
Lesotho	2 (<0.1)	16 (0.2)	0	18 (0.1)
Malawi	16 (0.3)	391 (5.1)	104 (1.0)	511 (2.2)
Mozambique	126 (2.3)	388 (5.1)	66 (0.7)	580 (2.5)
Namibia	19 (0.3)	196 (2.6)	48 (0.5)	263 (1.1)
South Africa	4,013 (72.4)	5,601 (73.1)	8,850 (87.6)	18,464 (79.2)
Zambia	426 (7.7)	182 (2.4)	84 (0.8)	692 (3.0)
Zimbabwe	232 (4.2)	329 (4.3)	98 (1.0)	659 (2.8)
Submitting country, no. (%)				
Botswana	83 (1.5)	216 (2.8)	799 (7.9)	1,098 (4.7)
Germany‡	1 (<0.1)	64 (0.8)	47 (0.5)	112 (0.5)
Malawi	14 (0.3)	8 (0.1)	38 (0.4)	60 (0.3)
South Africa	4,794 (86.5)	6,910 (90.2)	9,135 (90.4)	20,839 (89.4)
Spain‡	13 (0.2)	117 (1.5)	0	130 (0.6)
United Kingdom‡	210 (3.8)	189 (2.5)	0	399 (1.7)
United States‡	0	12 (0.2)	0	12 (0.1)
Zambia	426 (7.7)	144 (1.9)	84 (0.8)	654 (2.8)
Patient sex, no. (%)				
F	2,951 (53.2)	4,053 (52.9)	5,695 (56.4)	12,699 (54.5)
M	2,132 (38.5)	3,274 (42.7)	4,044 (40.0)	9,450 (40.5)
Unknown	460 (8.3)	333 (4.3)	364 (3.6)	1,157 (5.0)
Patient age, y, median (IQR)	37 (27–50)	37 (25–52)	39 (27–54)	38 (26–52)
Genomic surveillance coverage,† median (IQR)	1.55 (0.66–2.79)	3.76 (2.68–4.56)	3.98 (3.37–4.58)	3.48 (2.70–4.44)
Detected SARS-CoV-2 variants, no. (%)§				
Alpha, B.1.1.7 + Q.x	33 (0.6)	158 (2.1)	168 (1.7)	359 (1.5)
Beta, B.1.351 + B.1.351.x	749 (13.7)	6,176 (80.6)	1,493 (14.8)	8,418 (36.2)
Delta, B.1.617.2 + AY.x	0	129 (1.7)	7,454 (73.8)	7,583 (32.6)
Gamma, P.1 + P.1.x	1 (<0.1)	0	1 (<0.1)	2 (<0.1)
Variant of interest	0	1 (<0.1)	0	1 (<0.1)
Variant under monitoring	9 (0.2)	37 (0.5)	174 (1.7)	220 (0.9)
Former variant of interest	2 (<0.1)	1 (<0.1)	1 (<0.1)	4 (<0.1)
Lineages with no WHO label	4,673 (85.3)	1,155 (15.1)	812 (8.0)	6,640 (28.6)
January 2020 strain	14 (0.3)	3 (<0.1)	0	17 (0.1)

*Source: GISAID (https://www.gisaid.org), accessed 2021 Sep 20. IQR, interquartile range; World Health Organization. †Genomic surveillance coverage was defined as the number of reported cases per million per sample sequenced. A country with a higher number of reported cases per million per sample sequenced has lower genomic surveillance coverage than a country with a lower number of reported cases per million per sample sequenced. ‡Germany submitted sequences for specimens collected in Mozambique (n = 1), Namibia (n = 73), Zambia (n = 38). Spain submitted sequences for specimens collected in Mozambique (n = 130). UK submitted sequences for specimens collected in Zimbabwe (n = 401). The United States submitted sequences for specimens collected in South Africa (n = 12). §Specimens were classified using labels defined by the World Health Organization (Appendix Table 2).

**Table 4:** Country-level genomic surveillance comparison across 3 COVID-19 pandemic waves for 10 countries in southern Africa, March 1, 2020–September 6, 2021*

Country and collection period	No. samples† (% total)	In-country submissions (% samples)	Submitting country (% samples)	Genomic surveillance coverage,† median (IQR)	Patient sex (% samples)	Patient age, y, median (IQR)	SARS-CoV-2 variants detected (% samples)
Angola‡							
Before wk 2 start	615 (68.4)	0	South Africa (100.0)	0.98 (0.85–1.02)	M (55.9); F (43.4); Unk (0.7)	35.00 (21.25–47.00)	Alpha (5.2); Beta (31.1); Gamma (0.2); VUM (1.5); former VOI (0.3); other lineages (61.8)
After wk 2 start	264 (29.4)	0	South Africa (100.0)	2.71 (2.58–3.93)	M (61.0); F (38.6); Unk (0.4)	35.00 (24.00–45.00)	Alpha (40.5); Beta (25.4); Delta (4.2); VOI (0.4); VUM (10.6); other lineages (18.9)
After wk 3 start	20 (2.2)	0	South Africa (100.0)	58.02 (58.02–58.63)	M (25.0); F (75.0)	25.50 (7.75–34.75)	Alpha (5.0); Beta (10.0); Delta (85.0)
Overall	899 (100.0)	0	South Africa (100.0)	1.02 (0.94–2.53)	M (56.7); F (42.7); Unk (0.6)	35.00 (22.00–46.00)	Alpha (15.6); Beta (28.9); Delta (3.1); Gamma (0.1); VOI (0.1); VUM (4.1); former VOI (0.2); other lineages (47.8)
Botswana							
Before wk 2 start	83 (7.6)	83 (100.0)	Botswana (100.0)	48.22 (16.00–65.23)	F (49.4); M (31.3); Unk (19.3)	37.00 (25.50–44.00)	Beta (15.7); other lineages (84.3)
After wk 2 start, before wk 3 start	216 (19.7)	216 (100.0)	Botswana (100.0)	49.83 (37.96–67.59)	F (54.2); M (45.8)	35.00 (26.00–44.00)	Beta (85.6); Delta (1.4); other lineages (12.0); January 2020 strain (0.9)
After wk 3 start	799 (72.8)	799 (100.0)	Botswana (100.0)	37.30 (32.09–74.33)	F (52.7); M (46.9); Unk (0.4)	29.00 (15.00–41.00)	Beta (19.6); Delta (67.5); VUM (0.1); other lineages (12.8);
Overall	1,098 (100.0)	1,098 (100.0)	Botswana (100.0)	39.36 (33.02–71.40)	F (52.7); M (45.5); Unk (1.7)	31.00 (17.00–42.00)	Beta (32.3); Delta (49.4); VUM (0.1); other lineages (18.0); January 2020 strain (0.2)
Eswatini							
Before wk 2 start	11 (9.0)	0	South Africa (100.0)	483.33 (471.89–483.33)	F (63.6); M (36.4)	41.00 (24.50–49.00)	Beta (63.6); other lineages (36.4)
After wk 2 start, before wk 3 start	77 (63.1)	0	South Africa (100.0)	211.40 (191.60–211.40)	M (51.9); F (48.1)	35.00 (25.00–44.00)	Beta (24.7); Delta (67.5); VUM (3.9); other lineages (3.9)
After wk 3 start	34 (27.9)	0	South Africa (100.0)	493.27 (489.76–493.27)	M (58.8); F (41.2)	35.50 (28.50–42.50)	Beta (5.9); Delta (82.4); VUM (11.8)
Overall	33 (100.0)	0	South Africa (100.0)	211.40 (210.67–486.35)	M (52.5); F (47.5)	35.00 (25.25–44.00)	Beta (23.0); Delta (65.6); VUM (5.7); other lineages (5.7)
Lesotho							
Before wk 2 start	2 (11.1)	0	South Africa (100.0)	482.50 (479.55–485.45)	F (50.0); M (50.0)	25.00 (18.00–32.00)	Other lineages (100.0)
After wk 2 start, before wk 3 start	16 (88.9)	0	South Africa (100.0)	193.79 (92.81–203.15)	F (56.2); M (43.8)	47.00 (41.50–59.25)	Beta (87.5); Other lineages (12.5)
After wk 3 start	NA	NA	NA	NA	NA	NA	NA
Overall	18 (100.0)	0	South Africa (100.0)	203.15 (115.71–203.15)	F (55.6); M (44.4)	44.50 (39.25–55.75)	Beta (77.8); other lineages (22.2)
Malawi							
Before wk 2 start	16 (3.1)	14 (87.5)	Malawi (87.5); South Africa (12.5)	17.71 (12.97–18.36)	F (12.5); Unk (87.5)	30.00 (29.50–30.50)	Alpha (6.2); Beta (6.2); other lineages (87.5)
After wk 2 start, before wk 3 start	391 (76.5)	8 (2.0)	South Africa (98.0); Malawi (2.0)	2.71 (2.09–4.25)	M (60.1); F (37.3); Unk (2.6)	36.00 (26.00–46.00)	Alpha (1.0); Beta (83.6); Delta (6.6); other lineages (8.7)
After wk 3 start	104 (20.4)	38 (36.5)	South Africa (63.5); Malawi (36.5)	17.09 (17.02–19.45)	Unk (40.4); M (37.5); F (22.1)	37.00 (28.00–48.00)	Beta (4.8); Delta (84.6); c (10.6)
Overall	511 (100.0)	60 (11.7)	South Africa (88.3); Malawi (11.7)	4.14 (2.26–4.45)	M (53.6); F (33.5); Unk (12.9)	36.00 (26.00–46.75)	Alpha (1.0); Beta (65.2); Delta (22.3); other lineages (11.5)
Mozambique							
Before wk 2 start	126 (21.7)	0	South Africa (88.9); Spain (10.3); Germany (0.8)	3.28 (2.41–3.76)	M (55.6); F (44.4)	35.50 (29.00–46.25)	Beta (31.7); other lineages (68.3)
After wk 2 start, before wk 3 start	388 (66.9)	0	South Africa (69.8); Spain (30.2)	3.46 (2.00–5.10)	F (52.1); M (46.9); Unk (1.0)	33.00 (23.00–43.00)	Alpha (0.3); Beta (76.0); other lineages (23.7)
After wk 3 start	66 (11.4)	0	South Africa (100.0)	40.63 (39.15–43.29)	M (59.1); F (40.9)	32.00 (24.00–45.00)	Delta (100.0)
Overall	478 (100.0)	0	South Africa (77.4); Spain (22.4); Germany (0.2)	3.55 (2.10–5.13)	F (51.2); M (48.1); Unk (0.7)	34.00 (24.00–43.00)	Alpha (0.2); Beta (57.8); Delta (11.4); other lineages (30.7)
Namibia							
Before wk 2 start	19 (7.2)	0	South Africa (100.0)	263.12 (191.37–263.12)	F (57.9); M (42.1)	30.00 (25.50–36.00)	Other lineages (100.0)
After wk 2 start, before wk 3 start	196 (74.5)	0	South Africa (86.7); Germany (13.3)	76.60 (76.60–76.60)	F (60.7); M (39.3)	31.00 (18.00–43.00)	Alpha (1.5); Beta (55.1); other lineages (43.4)
After wk 3 start	48 (18.3)	0	Germany (97.9); South Africa (2.1)	724.01 (724.01–957.56)	F (62.5); M (37.5)	46.50 (32.00–62.75)	Beta (4.2); Delta (93.8); other lineages (2.1)
Overall	263 (100.0%)	0	South Africa (72.2); Germany (27.8)	76.60 (76.60–153.58)	F (60.8); M (39.2)	32.00 (19.00–47.00)	Alpha (1.1); Beta (41.8); Delta (17.1); other lineages (39.9)
South Africa							
Before wk 2 start	4,013 (21.7)	4,013 (100.0)	South Africa (100.0)	2.15 (0.70–2.83)	F (56.6); M (33.7); Unk (9.7)	37.00 (27.00–51.00)	Beta (11.4); other lineages (88.5); January 2020 strain (0.1)
After wk 2 start, before wk 3 start	5,601 (30.3)	5,589 (99.8)	South Africa (99.8); USA (0.2)	3.66 (2.71–4.51)	F (55.1); M (39.6); Unk (5.3)	39.00 (26.00–55.00)	Alpha (0.7); Beta (84.2); Delta (0.7); VUM (0.1); former VOI (0.0); other lineages (14.3)
After wk 3 start	8,850 (47.9)	8,850 (100.0)	South Africa (100.0)	3.85 (3.31–4.33)	F (57,6); M (39.4); Unk (3.0)	41.00 (28.00–55.00)	Alpha (1.9); Beta (15.0); Delta (73.4); Gamma (0.0); VUM (1.9); former VOI (0.0); other lineages (7.9)
Overall	18,464 (100.0)	18,452 (99.9)	South Africa (99.9); USA (0.1)	3.42 (2.84–4.28)	F (56.6); M (38.2); Unk (5.1)	39.00 (27.00–54.00)	Alpha (1.1); Beta (35.2); Delta (35.4); Gamma (0.0); VUM (0.9); former VOI (0.0); other lineages (27.3)
Zambia							
Before wk 2 start	426 (61.6)	426 (100.0)	Zimbabwe (100.0)	0.56 (0.23–0.79)	F (37.8); M (54.0); Unk (8.2)	39.00 (31.00–48.00)	Other variant (99.1); January 2020 strain (0.9)
After wk 2 start, before wk 3 start	182 (26.3)	144 (79.1)	Zimbabwe (79.1); Germany (20.9)	14.80 (6.82–19.88)	F (40.7); M (47.8); Unk (11.5)	32.00 (22.00–43.00)	Alpha (1.1); Beta (88.5); VUM (0.5); other lineages (9.3); January 2020 strain (0.5)
After wk 3 start	84 (12.1)	84 (100.0)	Zimbabwe (100.0)	59.11 (59.11–59.11)	F (39.3); M (57.1); Unk (3.6)	15.50 (14.25–17.00)	Delta (97.6); other lineages (2.4)
Overall	692 (100.0)	654 (94.5)	Zimbabwe (94.5); Germany (5.5)	0.89 (0.42–15.49)	F (38.7); M (52.7); Unk (8.5)	35.00 (23.00–46.00)	Alpha (0.3); Beta (23.3); Delta (11.8); VUM (0.1); other lineages (63.7); January 2020 strain (0.7)
Zimbabwe							
Before wk 2 start	232 (35.2)	0	UK (91.4); South Africa (8.6)	0.80 (0.06–3.39)	F (57.8); M (41.4); Unk (0.9)	34.50 (27.00–44.75)	Beta (20.4); other lineages (77.0); January 2020 strain (2.6)
After wk 2 start, before wk 3 start	329 (49.9)	0	UK (57.4); South Africa (42.6)	3.76 (2.74–4.89)	M (51.1); F (48.9)	38.00 (30.00–49.00)	Beta (86.3); other lineages (13.7)
After wk 3 start	98 (14.9)	0	South Africa (100.0)	53.33 (45.82–66.19)	Unk (55.1); F (22.4); M (22.4)	36.00 (29.50–46.00)	Beta (1.0); Delta (98.0); VUM (1.0)
Overall	659 (100.0)	0	UK (60.8); South Africa (39.2)	3.47 (2.65–5.62)	F (48.1); M (43.4); Unk (8.5)	37.00 (29.00–48.00)	Beta (50.5); Delta (14.6); VUM (0.9); other lineages (33.8); January 2020 strain (0.2)

*Source: GISAID (https://www.gisaid.org), accessed September 2021 Sep 20. IQR, interquartile range; NA, not applicable because Lesotho had no sequences submitted after the start of the third wave; VOI, variant of interest; VUM, variants under monitoring; Unk, unknown. †Genomic surveillance coverage is defined as the number of reported cases per sample sequenced. As such, a country with a higher number of reported cases per sample sequenced has lower genomic surveillance coverage than a country with a lower number of reported cases per sample sequences. ‡Angola was currently experiencing their third wave at the time of this analysis (September 19, 2021).

**Figure 3:**
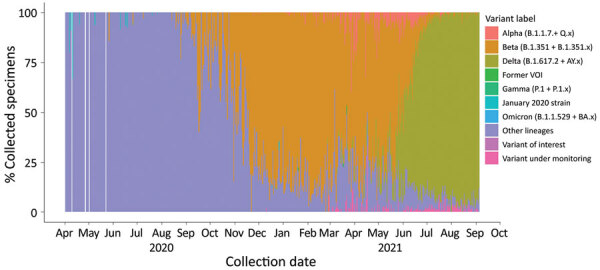
Percentage of SARS-CoV-2 variants among specimens submitted to GISAID in southern Africa, March 1, 2020–September 6, 2021. Definitions of variants are in Appendix Table 2. Source: GISAID (https://www.gisaid.org), accessed 2021 Sep 20.

Genomic surveillance coverage (median number of cases/1 million persons/SARS-CoV-2 genome submitted) varied across countries, from 1.02 (IQR 0.94–2.5)in Angola to 211.40 (IQR 210.7–486.4) in Eswatini (Table 4). For the southern Africa region, genomic surveillance coverage was highest before the start of the second wave, median 1.55 cases/1 million persons/SARS-CoV-2 genome submitted. The prevalence of the Beta variant increased from 13.7% in the period before the second wave to 80.6% during the second wave (Table 3). During the third wave, the prevalence of Beta decreased to 14.8% and the prevalence of Delta increased to 73.8%. Beta variant was predominant in the second wave in 8/10 countries and Delta in the third wave in 9/9 countries (Table 4; Figure 3).

### PHSMs

**Figure 5:**
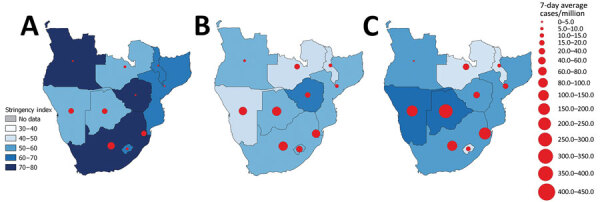
Comparison of public health and social measure stringency and 7-day average new COVID-19 cases per million across 3 COVID-19 pandemic waves in 10 countries in southern Africa, April 6, 2020–July 17, 2021. Source: GISAID (https://www.gisaid.org), accessed 2021 Sep 20.

PHSM stringency index decreased from the first through the third waves in 8/10 countries (Table 5; Figure 5). Regionally, average stringency, government response, and economic support indices were highest during the first wave (Table 5). International travel restrictions were the most common PHSM and closing public transport the least common (Table 6). During the first wave, more PHSMs were implemented at the beginning of the wave than at the end, whereas during the second wave, more PHSMs were implemented at the end of the wave than the beginning. For all 3 waves, the most PHSMs were implemented at the peak of the wave (Table 7).

**Table 5:** Public health and social measure comparison across COVID-19 pandemic waves for 10 countries in southern Africa, January 1, 2020–September 19, 2021*,

Country and wave	Average index number throughout entire wave					No. stringent containment measures‡ in place†			
	Stringency index‡	Government response index§	Containment index¶	Economic support index#		Beginning of wave	Peak of wave	End of wave	Entire wave
Angola									
1	69.7	50.88	56.22	13.49		8	8	8	8
2	49.5	43.53	49.74	0.00		8	5	7	5
3	56.9	48.01	54.86	0.00		7	Unknown	7	7
Overall	57.1	42.79	48.31	12.67		NA	NA	NA	NA
Botswana									
1	57.5	49.75	51.03	40.79		8	7	7	7
2	59.2	51.60	57.94	7.14		7	7	7	7
3	60.3	58.08	65.22	8.13		7	7	4	0
Overall	54.7	46.97	50.52	22.15		NA	NA	NA	NA
Eswatini									
1	72.9	54.39	58.58	25.00		8	8	8	8
2	67.6	59.52	60.17	55.00		7	8	7	7
3	63.7	64.42	73.32	2.27		8	8	8	8
Overall	59.1	50.93	53.99	29.50		NA	NA	NA	NA
Lesotho									
1	62.6	49.28	49.86	45.17		7	8	7	6
2	62.7	49.24	56.27	0.00		7	6	8	6
3	54.2	50.18	57.35	0.00		5	6	6	5
Overall	53.3	42.99	46.98	13.91		NA	NA	NA	NA
Malawi									
1	59.8	51.42	51.11	53.57		6	6	6	6
2	49.0	49.08	50.17	41.45		5	6	5	4
3	40.5	48.04	51.42	24.33		4	6	4	4
Overall	44.8	42.97	43.99	35.83		NA	NA	NA	NA
Mozambique									
1	70.5	55.22	63.11	0.00		8	8	6	5
2	56.5	48.16	55.04	0.00		5	7	7	5
3	52.2	47.63	54.43	0.00		6	7	6	5
Overall	52.9	43.76	50.01	0.00		NA	NA	NA	NA
Namibia									
1	54.4	49.31	53.05	23.13		7	7	6	5
2	38.8	38.75	44.48	0.00		6	5	6	4
3	57.4	56.26	64.20	0.69		7	8	6	4
Overall	46.6	43.25	48.23	8.96		NA	NA	NA	NA
South Africa									
1	79.4	74.51	77.12	56.21		8	8	7	7
2	53.2	63.18	61.49	75.00		5	8	7	5
3	52.7	60.73	62.22	50.18		6	8	7	3
Overall	54.0	57.53	58.14	53.19		NA	NA	NA	NA
Zambia									
1	49.1	43.76	46.44	25.00		6	6	6	5
2	41.8	38.25	42.27	10.06		6	6	4	3
3	45.7	42.87	46.44	17.86		3	5	4	1
Overall	40.6	36.60	39.56	15.88		NA	NA	NA	NA
Zimbabwe									
1	77.8	58.05	62.77	25.00		8	8	8	8
2	80.3	61.45	66.96	2.86		8	8	6	6
3	67.0	55.20	63.08	0.00		5	6	8	5
Overall	2.9	48.78	53.88	13.06		NA	NA	NA	NA
Total averages per wave									
1	65.4	53.66	56.93	30.74		7.4	7.5	6.9	6.5
2	55.9	50.27	54.45	21.15		6.4	6.6	6.4	5.2
3	55.1	53.14	59.25	10.35		5.8	6.7	6.0	4.2
Overall	52.6	45.76	49.36	20.51		NA	NA	NA	NA

*Public health and social measures, as defined and measured by Oxford COVID-19 Government Response Tracker (OxCGRT; https://www.bsg.ox.ac.uk/research/research-projects/covid-19-government-response-tracker), accessed 2021 Sep 20. OxCGRT collects publicly available information on 23 indicators of government response. The data from these indicators is aggregated into a set of common indices, reporting a number between 1 and 100 to reflect the level of government action in 4 specific areas. This is a measure of how many of the relevant indicators a government has acted upon, and to what degree. A higher number reflects more government action. NA, not applicable. †For this analysis, we focused on 8 indicators of stringent containment measures, which record information on containment and closure policies such as school closures and restrictions in movement. If any containment measure related to that indicator was in place at the time of the wave that is indicated, the measure was counted. ‡Stringency index records the strictness of ‘lockdown style’ policies that primarily restrict residents’ behavior. It is calculated using all ordinal containment and closure policy indicators, plus an indicator recording public information campaigns. §Overall government response index records how the response of governments has varied over all indicators in the database, becoming stronger or weaker over the course of the outbreak. It is calculated using all ordinal indicators. ¶Containment and health index combines ‘lockdown’ restrictions and closures with measures such as testing policy and contact tracing, short term investment in healthcare, as well investments in vaccines. It is calculated using all ordinal containment and closure policy indicators and health system policy indicators. #Economic support index records measures such as income support and debt relief. It is calculated using all ordinal economic policies indicators.

**Table 6:** Most frequent public health and social measure types implemented across COVID-19 pandemic waves for 10 countries in southern Africa, January 1, 2020–September 19, 2021*

Wave	Most common measures	Least common measures
Wave 1		
Beginning	Workplace closing, cancel public events, restrictions on gatherings, international travel	Public transport closings
Peak	School closing, workplace closing, cancel public events, restrictions on gatherings, international travel	Public transport closings
End	School closing, workplace closing, cancel public events, restrictions on gatherings, international travel	Public transport closings
Duration	School closing, workplace closing, cancel public events, international travel	Public transport closings
Wave 2		
Beginning	Workplace closing, cancel public events, restrictions on gatherings, international travel	Public transport closings
Peak	School closing, workplace closing, cancel public events, restrictions on gatherings, international travel	Movement restrictions
End	School closing, cancel public events, restrictions on gatherings, international travel	Close public transport, movement restrictions
Duration	School closing, cancel public events, restrictions on gatherings, international travel	Public transport closings
Wave 3		
Beginning	Restrictions on gatherings, international travel	Movement restrictions
Peak	Workplace closing, cancel public events, restrictions on gatherings, international travel	Public transport closings, movement restrictions
End	Workplace closing, international travel	Movement restrictions
Duration	International travel	School closings

*Public health and social measures, as defined and measured by Oxford COVID-19 Government Response Tracker (https://www.bsg.ox.ac.uk/research/research-projects/covid-19-government-response-tracker), accessed 2021 Sep 20.

**Table 7:** Number of public health and social measures implemented across COVID-19 pandemic waves by type for 10 countries in southern Africa, January 1, 2020–September 19, 2021*

Intervention	Wave 1, n = 10					Wave 2, n = 10					Wave 3, n = 10†			
	Start	Peak	End	Duration		Start	Peak	End	Duration		Start	Peak	End	Duration
School closings	9	10	10	10		9	10	10	10		4	7*	2	1
Workplace closings	10	10	10	10		10	10	9	9		9	9*	10	6
Canceled public events	10	10	10	10		10	10	10	10		9	9*	9	7
Restrictions on gatherings	10	10	10	9		10	10	10	10		10	9*	9	8
Public transport closings	7	6	4	4		2	5	5	0		5	5*	7	2
Stay-at-home requirements	9	9	8	7		7	9	10	6		8	7*	8	7
Movement restrictions	9	9	7	6		6	4	5	2		3	5*	4	2
International travel	10	10	10	10		10	10	10	10		10	9*	10	9

*Public health and social measures, as defined and measured by Oxford COVID-19 Government Response Tracker https://www.bsg.ox.ac.uk/research/research-projects/covid-19-government-response-tracker), accessed 2021 Sep 20. †For peak of wave 3, n = 9.

### Vaccination Coverage

Countries began SARS-CoV-2 vaccination campaigns after the first wave during February 17 (South Africa) through April 14, 2021 (Zambia) (*5*). By the time the second wave began, 7/10 countries (excluding Namibia, Lesotho, and Eswatini) had begun vaccinations; all countries had begun vaccinations by the third wave (Table 2). As of September 19, 2021, 10.8% of the population was vaccinated on average across southern Africa and 8.1% fully vaccinated (Table 2). Coverage varied by country: Eswatini had 16.5% and Zambia 1.5% fully vaccinated. Seven-day vaccinations per 1 million persons steadily increased across waves and were 4.2-fold higher during the third wave (1,087.9) than the second (262.1) (Table 2).

## Discussion

Among key findings, we found that patterns of wave propagation throughout the region were similar across almost all country waves. In the absence of a representative regional surveillance system for influenza-like illness, surveillance data from South Africa, where waves were first detected, provided an early warning signal for other countries in the region. Although per person volume of testing increased over time in southern Africa, it remained low compared with resource-rich countries and differed among countries, limiting the ability to compare reported incidence and mortality. Genomic sequencing in the region was limited outside of South Africa. In most countries, reported percentage positivity, incidence rates, mortality rates, and CFRs increased across waves, partly caused by the emergence of more transmissible variants. Stringent PHSM implementation declined over successive waves, and vaccine coverage was low.

Because South Africa accounted for >30% of cases in Africa, average wave patterns were similar between southern Africa and Africa as a whole but with notable regional and intercountry variations (*6*). Kenya, in eastern Africa, experienced second and third waves before southern Africa. In southern Africa, all waves followed a similar regional pattern: waves were first detected in South Africa, then throughout the remaining interconnected countries an average of 7.2 weeks later. This pattern was less obvious for Angola, where the second wave started 20 weeks after South Africa (Appendix Table 1). This pattern likely reflects greater testing capacity in South Africa, more sensitive surveillance, and possibly mobility characteristics in the region because South Africa is an international transportation hub. According to phylogenetic analysis, South Africa was determined to be the source of SARS-CoV-2 cases imported to the rest of the region during the first and second pandemic waves (*17*). Awareness of this pattern is critical for future mitigation efforts; pretravel testing and ongoing sentinel surveillance might be critical for detecting cross-border transmission early, and pandemic surveillance and reporting in South Africa can serve as an early warning signal for countries with more limited testing capacity. However, a regional, representative, surveillance system for influenza-like illness and severe acute respiratory illness could improve regional detection and response systems.

Although weekly population-level numbers of tests increased, testing per case, an indicator of sufficient coverage in high-transmission periods, decreased across waves, and the region never achieved the WHO-recommended target of 1,000 tests/1 million persons (*18*). The region’s average testing volume per person was low compared with resource-rich countries: ≈240 tests/1 million persons/day in southern Africa versus >3,000 tests/1 million/day in the United States (*4*). Even Namibia and South Africa, despite relatively higher testing volumes, were below the WHO target for testing. This target might be unreachable for most countries in this region unless test accessibility for the general population is substantially improved. Increasing availability and feasibility of COVID-19 self-tests, as recommended by Africa CDC (*19*), might increase testing and improve public health mitigation efforts (*20*,*21*).

In this resource-constrained region, testing volumes should be expanded on the basis of need and be designed to collect data to address key objectives for public health response. These data include diagnosing admissions, classifying excess deaths because of COVID-19, defining the timing of pandemic waves, monitoring circulating variants, and informing guidance for work, school, and social engagements. Data gathered from serosurveillance and postmortem activities might also help address these objectives (*3*,*4*,*22*).

Our ability to directly compare SARS-CoV-2 case and death counts in the region using publicly available data was limited by changes over time in test types and availability, low likelihood of diagnosis (*4*), and various and changing testing strategies. Sustaining COVID-19 sentinel surveillance systems in the community and among high-risk populations (*23*), including through targeted use of antigen rapid diagnostic tests (*24*), and improving standard reporting throughout the region to ensure appropriate local epidemiologic evaluations and responses (*25*), could be considered. These data-gathering systems could be coordinated through a regional body such as the recently established Africa CDC Southern Africa Regional Collaborating Centre (*26*).

Genomic sequencing varied across countries and was limited outside South Africa. Low sequencing limits detection of new VOCs, posing regional and global health security risks. Africa CDC and WHO are strengthening genomic surveillance by establishing a continentwide laboratory network, leveraging existing surveillance systems, to better detect variant evolution (*27*). To improve sequencing of SARS-CoV-2 and other endemic and epidemic pathogens, systematic in-country genomic surveillance could be built and sustained in the region by adopting sequencing targets such as weekly targets based on incidence and estimated prevalence of variants in line with Africa CDC guidelines (*28*–*30*). In southern Africa, Beta variant was predominant in the second wave and Delta in the third.

Across the region, the third COVID-19 wave had the highest 7-day average percentage positivity, daily cases, deaths per 1 million population, and CFR. Increases in reported incidence and mortality at a time of increasing percentage positivity occurred at least partly because of the emergence of more transmissible variants across waves. However, the connection between high testing volume and reported incidence and mortality rates per person in upper-middle-income countries Namibia and South Africa might reflect better testing capacity contributing to improved accuracy of identifying cases and classifying cause of death, leading to higher reported overall incidence and mortality rates (*31*).

Neither emergence of more transmissible variants nor improved testing capacity can fully explain the increase in CFR over time, an observation that has been previously reported for Africa (*32*–*34*). Possible explanations for this increase include increased strain on limited critical care capacity as transmission and hospitalizations increased (*6*,*34*,*35*); health systems with minimal critical care resources are not optimized for managing critically ill COVID-19 cases. A recent prospective cohort analysis found that mortality among critically ill hospitalized patients was 48.2% in Africa, higher than the estimated 31.5% global average (*32*,*34*). Other explanations might include delays in healthcare-seeking behavior by patients, improved differential testing and reporting (i.e., relatively fewer tests among persons who are not ill but more among very ill persons), improvements in classifying COVID-19–related deaths, and declining ability to protect vulnerable populations from SARS-CoV-2 exposure. However, increased CFR in the region suggests the need for improved health systems and access to newer therapeutics for high-risk patients, such as antivirals molnupiravir (*36*) and nirmatrelvir (*37*).

The increased incidence, mortality, and CFR during the third wave were not universal across countries. Lesotho reported highest average incidence rates during its second wave, and Lesotho and South Africa reported highest average mortality rates during the second waves. Eswatini also reported a lower CFR in its third wave than in its second. Possible explanations for those patterns include development of natural immunity to severe disease (*4*), improved outbreak response and service delivery (*38*), or incomplete data analysis because the third wave was not yet complete when we collected data.

Declining stringency in adherence to PHSMs in the region likely occurred as governments acknowledged sociopolitical, cultural, and economic context, rather than just epidemiologic data, to determine appropriate restrictions (*39*). Decreasing acceptance of and adherence to PHSMs has been observed in 4 countries in the region, in part because of negative effects on livelihoods and lack of access to health services (*23*). To improve adherence, PHSMs could be introduced, adapted, and lifted based on situational assessments in each country and considering community feedback (*25*,*40*,*41*). Given likely challenges in implementing and enforcing stringent PHSMs in the future, policymakers could consider targeting new measures towards persons at highest risk for severe disease.

On average, 8.1% of the population in the region was fully vaccinated as of September 19, 2021, compared with 46.7 in Morocco, 53.9 in the United States, and 63.6 in Israel (*5*). Vaccine coverage in southern Africa faced challenges including low domestic manufacturing capacity, donations of vaccines near their expiration dates, vaccine hoarding by high-income countries, and low vaccine uptake (*42*,*43*), highlighting the need to expand equitable access to vaccines and regional vaccine manufacturing capacity (*44*). Considering the WHO-recommended target that 70% of the population be fully vaccinated by mid-2022 might be unrealistic for the region (*45*) and likely high SARS-CoV-2 seropositivity (*4*), vaccination campaigns targeting populations in the region at highest risk for death, such as persons who are elderly or have chronic underlying conditions (*46*), might be effective in reducing severe disease and emergence of VOCs (*47*). To expand access to COVID-19 vaccinations, particularly for immunosuppressed persons, some countries in Africa (e.g., Zambia) have integrated COVID-19 vaccination services into existing health delivery platforms and clinics (e.g., HIV clinics); bringing vaccine access closer to home might aid in uptake (*48*).

We used publicly available datasets, each with data quality challenges. The OWID dataset missed some daily reports, so we requested coauthor data validation from country officials and Africa CDC to ensure reliability of the data. However, missing data from OWID limited our ability to compare pandemic waves between countries, especially those outside South Africa. OWID uses date reported, rather than specimen collection date, meaning that waves might have appeared to begin and end later in countries with time lags between testing and reporting. We assumed standard WHO definitions were used for reporting COVID-19 cases and deaths in the OWID dataset. We did not account for changes in test availability and testing strategies over time, which limited consideration of potential differences in those indicators among countries. The GISAID dataset varied in representativeness because some countries submitted very limited specimens, so we reported genomic surveillance results at a country level to highlight variability among countries. The OxCGRT dataset includes safety and control measures mandated by governments but not the extent of adherence to the measures, which might better correlate with transmission. Regional trends might be more influenced by data reported by an individual country, particularly South Africa, which provided most OWID and GISAID data. Our data were also extracted while the third wave was ongoing in the region, although it was declining except in Angola, where the third wave had not yet peaked by September 19, 2021. Despite those limitations, by soliciting data reviews from representatives for each country, reporting results at a country level, and computing regional indicators averaging country rates adjusted for population size and daily variation, we have compiled a reasonable description of the pandemic situation across southern Africa.

By September 19, 2021, southern Africa had experienced 3 waves of COVID-19, almost all first detected in South Africa, and with successively higher reported percentages of positivity, incidence, mortality, and CFRs. Increased incidence and mortality could be partly explained by the emergence of more transmissible SARS-CoV-2 variants and improved testing capacity and surveillance. Increasing CFRs warrants further research and highlights opportunities for strengthening health systems and increasing access to feasible therapeutics for high-risk persons. Testing volume increased across waves but varied by country and remained low compared with resource-rich countries. Genomic surveillance capacity was limited, although South Africa played a key role in supporting other countries. Stringent PHSM implementation declined over time, indicating a decrease in feasibility. Vaccination coverage remained very low; scale-up, especially among high-risk persons, should be considered. Coordinated regional solutions could be considered to strengthen and sustain sentinel surveillance systems, genomic surveillance capacity, risk-based vaccination, and tailored public health mitigation to better detect, prevent, and reduce the severity of future COVID-19 waves and other outbreaks in southern Africa.

Appendix Additional information about comparison of COVID-19 pandemic waves in 10 countries in southern Africa during 2020–2021.
