# Supplementary material for: Comparison of COVID-19 Pandemic Waves in 10 Countries in Southern Africa, 2020–2021
Source: Emerg Infect Dis. 2022 Dec;28(Suppl 1):S93–S104. doi: 10.3201/eid2813.220228 (PMC9745237; doi:10.3201/eid2813.220228)
Supplement: Appendix — Additional information about comparison of COVID-19 pandemic waves in 10 countries in southern Africa during 2020–2021. [file 22-0228-Techapp-s1.pdf]

Article DOI: https://doi.org/10.3201/eid2813.220228

Comparison of COVID-19 Pandemic Waves in 10 Countries in Southern Africa, 2020–2021

Appendix

Appendix Table 1. Dates for starts, ends and peaks of COVID-19 pandemic waves in 10 Southern African countries, 06 April 2020 to 19 September 2021*

	First wave			Second wave			Third wave†	
Country	Start	Peak	End	Start	Peak	End	Start	Peak
Angola	06 July 2020 (week 28)	25 Oct 2020 (week 43)	07 Feb 2021 (week 58)	29 March 2021 (week 66)	30 May 2021 (week 74)	04 July 2021 (week 79)	05 July 2021 (week 80)	-
Botswana	11 May 2020 (week 20)	18 Oct 2020 (week 42)	20 Dec 2020 (week 51)	04 Jan 2021 (week 54)	07 March 2021 (week 62)	02 May 2021 (week 70)	17 May 2021 (week 73)	08 Aug 2021 (week 84)
Eswatini	22 June 2020 (week 26)	16 Aug 2020 (week 33)	11 Oct 2020 (week 41)	07 Dec 2020 (week 50)	24 Jan 2021 (week 56)	21 March 2021 (week 64)	05 July 2021 (week 80)	15 Aug 2021 (week 85)
Lesotho	22 June 2020 (week 26)	02 Aug 2020 (week 31)	18 Oct 2020 (week 42)	30 Nov 2020 (week 49)	10 Jan 2021 (week 54)	28 Feb 2021 (week 61)	07 June 2021 (week 76)	01 Aug 2021 (week 83)
Malawi	15 June 2020 (week 25)	12 July 2020 (week 28)	04 Oct 2020 (week 40)	14 Dec 2020 (week 51)	24 Jan 2021 (week 56)	25 April 2021 (week 69)	31 May 2021 (week 75)	25 July 2021 (week 82)
Mozambique	29 June 2020 (week 27)	20 Sept 2020 (week 38)	22 Nov 2020 (week 47)	21 Dec 2020 (week 52)	31 Jan 2021 (week 57)	09 May 2021 (week 71)	24 May2021 (week 74)	01 Aug 2021 (week 83)
Namibia	15 June 2020 (week 25)	23 Aug 2020 (week 34)	08 Nov 2020 (week 45)	09 Nov 2020 (week 46)	27 Dec 2020 (week 52)	14 Feb 2021 (week 59)	17 May 2021 (week 73)	27 June 2021 (week 78)
South Africa	06 April 2020 (week 15)	19 July 2020 (week 29)	13 Sept 2020 (week 37)	09 Nov 2020 (week 46)	10 Jan 2021 (week 54)	07 March 2021 (week 62)	03 May 2021 (week 71)	04 July 2021 (week 79)
Zambia	06 July 2020 (week 28)	02 Aug 2020 (week 31)	25 Oct 2020 (week 43)	30 Nov 2020 (week 49)	17 Jan 2021 (week 55)	02 May 2021 (week 70)	17 May 2021 (week 73)	27 June 2021 (week 78)
Zimbabwe	29 June 2020 (week 27)	02 Aug 2020 (week 31)	20 Sept 2020 (week 38)	28 Dec 2020 (week 53)	10 Jan 2021 (week 54)	07 March 2021 (week 62)	07 June 2021 (week 76)	18 July 2021 (week 81)

Source: Our World in Data (OWID), accessed 20 September 2021

*We used Salyer et al.'s start week for a country's first wave. Otherwise, 'rising numbers of COVID-19 cases' were classified by examining fold-increases in weekly cases per million, where the first week of at least 1-fold sequential week-by-week increases indicated the start week of a wave . Peak weeks were defined as a local maximum preceded by sequential week-by-week increases and followed by a sequential week-by-week decline. End weeks were defined as the first week of a local minimum following a sequential week-by-week decline. To align with global epidemiologic reporting, we used WHO epidemiologic weeks: start dates are the first day of that week (Monday); and end dates are the last day of that week (Sunday). Given the selected definition for start, peak and end weeks, wave periods used for this analysis and therefore resultant statistics may differ from those used in-country by Southern African governments that may have applied a different definition for wave start and end weeks.

†We used 19 Sept 2021 (week 90), the final date of data extraction for our study, as the end date of the third wave for all southern Africa countries although the wave was ongoing in Angola

.

Appendix Table 2. World Health Organization (WHO) label classification of SARS-CoV-2 variants by Pango lineage

WHO label	Pango lineage
Alpha	B.1.1.7 + Q.x
Beta	B.1.351 + B.1.351.x
Gamma	P.1 + P.1.x
Delta	B.1.617.2 + AY.x
Variants of interest (VOI)	C.37 (Lambda); B.1.621 (Mu)
Variants under monitoring (VUM)	B.1.427; B.1.429; R.1; B.1.466.2; B.1.1.318; B.1.1.519; C.36.3; B.1.214.2; B.1.1.523; B.1.619; B.1.620; C.1.2; B.1.617.1 (Kappa); B.1.526 (Iota); B.1.525 (Eta); B.1.429 (Epsilon)
Former variant of interest (VOI)*	P.2 (Zeta); P.3 (Theta)
Other lineages	All other lineages
January 2020 strain	A

Source: WHO variant label classification (https://www.who.int/en/activities/tracking-SARS-CoV-2-variants), accessed 20 September 2021
*No longer a VUM or VOI

Appendix Table 3. Distribution of SARS-CoV-2 lineages representing >1% of other lineages classification, 01 March 2020–19 September 2021

Lineage classification in GISAID	Number of sequences	Other lineages category sequences, %
None*	1,337	20.1
B.1	1,031	15.5
B.1.1	571	8.6
B.1.1.448	467	7.0
C.1	383	5.8
B.1.1.54	297	4.5
B.1.237	202	3.0
C.16	187	2.8
B.1.1.273	149	2.2
B.1.1.412	129	1.9
B.1.381	121	1.8
B.1.1.528	89	1.3
AE.1	83	1.3
B.1.1.57	75	1.1
B.1.1.117	73	1.1
B.1.1.34	71	1.1
B.1.1.52	71	1.1
B.1.1.111	66	1.0
Total†	5402	81.4

Source: Global Initiative on Sharing Avian Influenza Data (GISAID), accessed 20 September 2021
*No lineage was specified in the GISAID database, although a clade was specified for these specimens.
†There were a total of 6,640 sequences during the period. The displayed 5,402 sequences represented 81.4% total lineages that composed at least 1.0% each of the other lineages classification

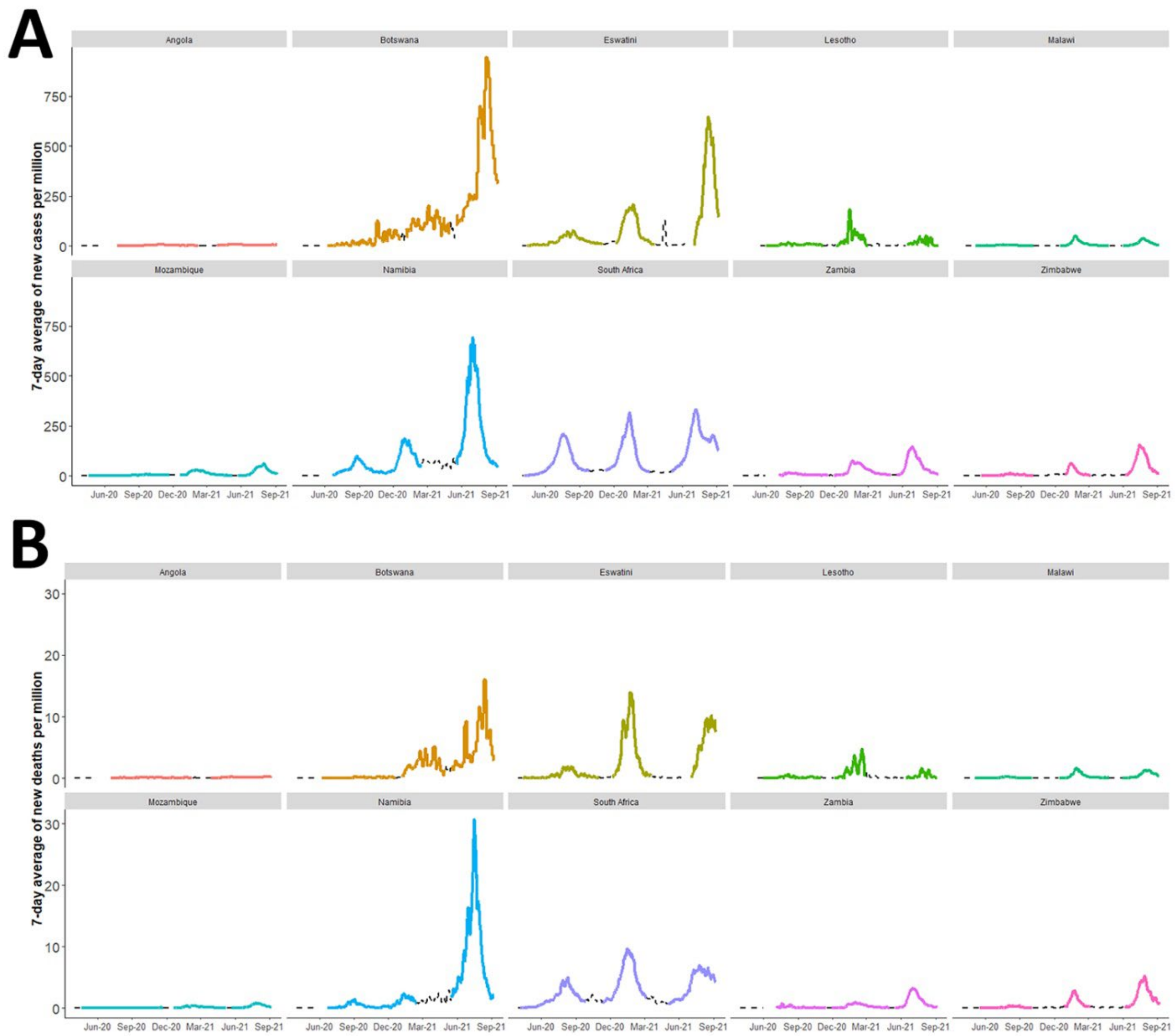

Appendix Figure 1. Reported 7-day average A) new COVID-19 cases and COVID-19 deaths /1 million persons across pandemic waves in 10 southern Africa countries, 05 March 2020 – 19 September 2021. Colored lines indicate designated wave periods, dashed lines indicate interwave periods. Corresponding Y axes scales were used in this figure to better visualize the comparison of wave magnitudes across countries. Source: Our World in Data (OWID).

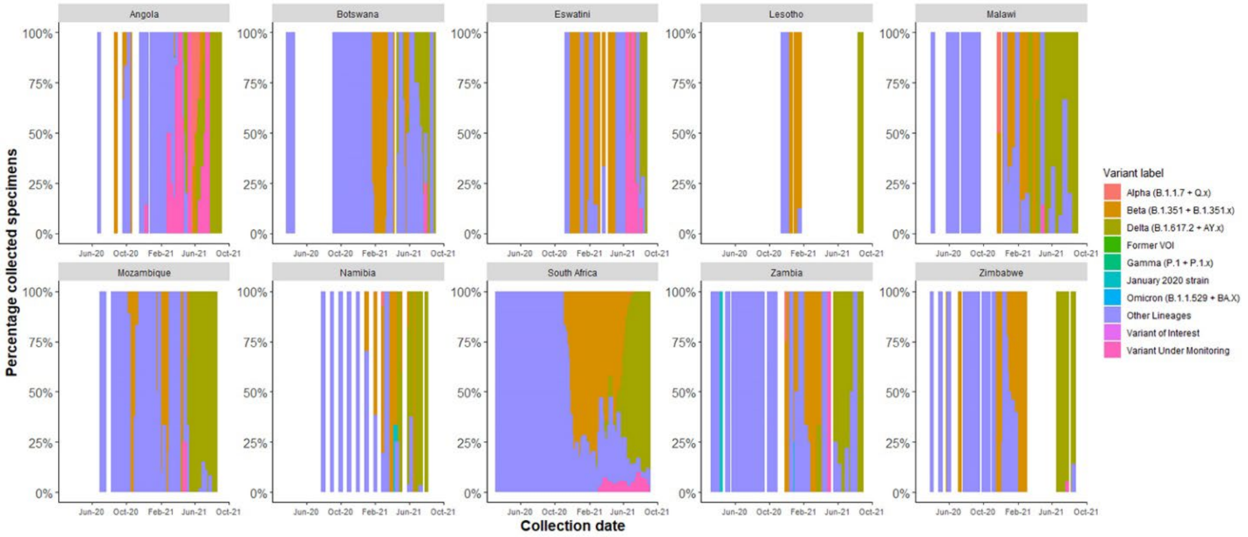

Appendix Figure 2. Percentage of collected SARS-CoV-2 specimens by country submitting to GISAID across 10 southern Africa countries, 1 March 2020 – 6 September 2021 (collection date). Source: Global Initiative on Sharing Avian Influenza Data (GISAID).

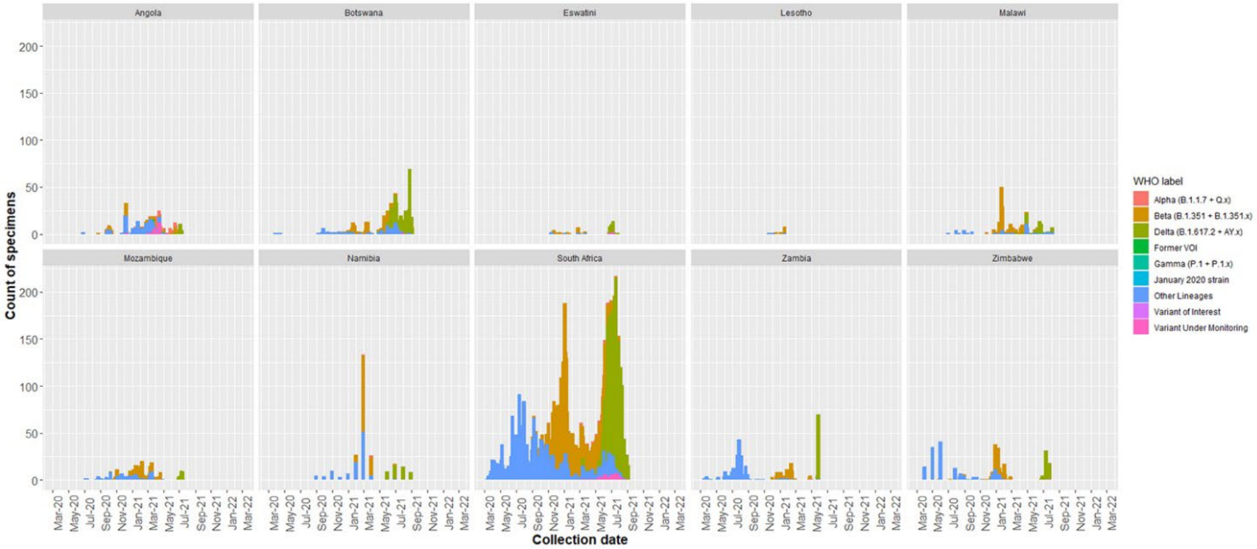

Appendix Figure 3. Counts of SARS-CoV-2 variants in 10 southern African countries, 1 March 2020 – 6 September 2021 (collection date). Variants were classified according to World Health Organization labels (Appendix Table 2). Corresponding Y axes scales were used in this figure to better visualize the comparison of genomic sampling magnitudes across countries. Source: Global Initiative on Sharing Avian Influenza Data (GISAID).
